# Functional connectivity in older adults—the effect of cerebral small vessel disease

**DOI:** 10.1093/braincomms/fcad126

**Published:** 2023-04-19

**Authors:** Nadieh Drenth, Jessica C Foster-Dingley, Anne Suzanne Bertens, Nathaly Rius Ottenheim, Roos C van der Mast, Serge A R B Rombouts, Sanneke van Rooden, Jeroen van der Grond

**Affiliations:** Department of Radiology, Leiden University Medical Center, P.O. Box 9600, 2300 RC Leiden, The Netherlands; Department of Radiology, Leiden University Medical Center, P.O. Box 9600, 2300 RC Leiden, The Netherlands; Department of Psychiatry, Leiden University Medical Center, P.O. Box 9600, 2300 RC Leiden, The Netherlands; Department of Radiology, Leiden University Medical Center, P.O. Box 9600, 2300 RC Leiden, The Netherlands; Department of Psychiatry, Leiden University Medical Center, P.O. Box 9600, 2300 RC Leiden, The Netherlands; Department of Psychiatry, Leiden University Medical Center, P.O. Box 9600, 2300 RC Leiden, The Netherlands; Department of Psychiatry, Leiden University Medical Center, P.O. Box 9600, 2300 RC Leiden, The Netherlands; Department of Psychiatry, Collaborative Antwerp Psychiatric Research Institute (CAPRI)–University of Antwerp, Antwerp, Belgium; Department of Radiology, Leiden University Medical Center, P.O. Box 9600, 2300 RC Leiden, The Netherlands; Institute of Psychology, Leiden University, P.O. Box 9555, 2300 RB Leiden, The Netherlands; Leiden Institute for Brain and Cognition, P.O. Box 9600, 2300 RC Leiden, The Netherlands; Department of Radiology, Leiden University Medical Center, P.O. Box 9600, 2300 RC Leiden, The Netherlands; Department of Radiology, Leiden University Medical Center, P.O. Box 9600, 2300 RC Leiden, The Netherlands

**Keywords:** functional connectivity, resting state networks, graph theory, ageing, cerebral small vessel disease

## Abstract

Ageing is associated with functional reorganization that is mainly characterized by declining functional connectivity due to general neurodegeneration and increasing incidence of disease. Functional connectivity has been studied across the lifespan; however, there is a paucity of research within the older groups (≥75 years) where neurodegeneration and disease prevalence are at its highest. In this cross-sectional study, we investigated associations between age and functional connectivity and the influence of cerebral small vessel disease (CSVD)—a common age-related morbidity—in 167 community-dwelling older adults aged 75–91 years (mean = 80.3 ± 3.8). Resting-state functional MRI was used to determine functional connectivity within ten standard networks and calculate the whole-brain graph theoretical measures global efficiency and clustering coefficient. CSVD features included white matter hyperintensities, lacunar infarcts, cerebral microbleeds, and atrophy that were assessed in each individual and a composite score was calculated. Both main and interaction effects (age*CSVD features) on functional connectivity were studied. We found stable levels of functional connectivity across the age range. CSVD was not associated with functional connectivity measures. To conclude, our data show that the functional architecture of the brain is relatively unchanged after 75 years of age and not differentially affected by individual levels of vascular pathology.

## Teaser

Drenth et al. investigated age effects and the influence of common age-related vascular pathology on functional connectivity in adults aged 75 years and older. The authors show that the brain’s functional architecture is relatively unchanged after 75 years of age and not differentially affected by individual levels of vascular pathology.

## Graphical Abstract

**Graphical abstract:**
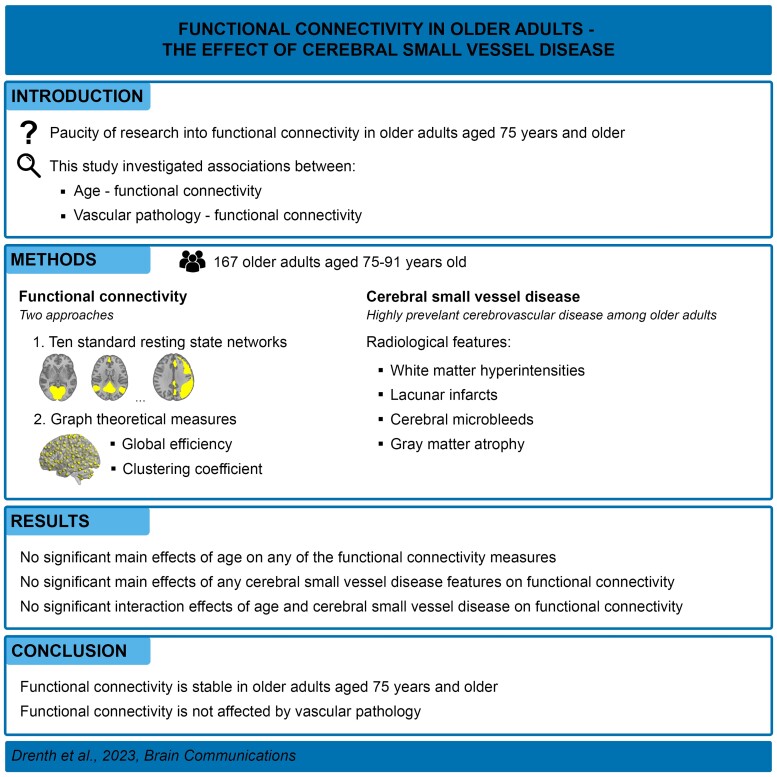

See Schreiber and Arndt (https://doi.org/10.1093/braincomms/fcad135) for a scientific commentary on this article.

## Introduction

Brain function is supported by the intrinsic functional organization of the brain. One way to study the functional organization of the brain is through intrinsic functional connectivity. Functional connectivity can be mapped by correlating regional spontaneous functional magnetic resonance imaging (fMRI) signal obtained while the subject is at rest, i.e. not performing any task.^1,2^ Several so called ‘resting state networks’ (RSNs) of highly synchronized brain regions have been identified and associated with diverse sets of brain functions such as sensory processing or cognitive function.^3–6^ In many neurodegenerative diseases, this functional organization of the brain is disrupted, often already in an early stage of the disease.^7–9^ Even in relatively healthy ageing, age-related changes in functional connectivity can be identified and related to (beginning) functional deficits such as cognitive dysfunction.^10–14^ Functional reorganization shows a general ageing trend of a loss of brain segregation as functional connectivity in large-scale networks decreases with advancing age.^11,15^ Functional connectivity alterations appear to be most pronounced in the oldest adults,^11,14^ although there is a scarcity of research in people aged 75 years and older. At the same time, this group is at greatest risk of functional decline due to general neurodegenerative processes and increased prevalence of neurodegenerative and cerebrovascular disease. Therefore, it is of particular interest to investigate age-related alterations in functional connectivity at older age and examine which age-related morbidities may specifically affect functional connectivity.^15^

One of the most common manifestations of age-related morbidity is the occurrence of cerebral small vessel disease (CSVD). CSVD is highly prevalent amongst older people and considered a major cause of vascular cognitive impairment, Alzheimer’s disease, dementia, and associated with increased risk of stroke, depression, and mortality.^16,17^ The main risk factors for CSVD are hypertension and ageing. In CSVD, vessel wall damage of the small vessels in the brain lead to focal lesions including lacunar infarcts, cerebral microbleeds (CMBs), and white matter hyperintensities (WMHs).^16,18^ It has been suggested that these focal lesions result in a wide variability of functional deficits in part through its interference on functional connectivity between brain regions.^19^ Nevertheless, only few studies to date have investigated functional connectivity in CSVD and findings are inconsistent. Decreased functional connectivity in CSVD patients has been reported,^20^ whilst there is also evidence that CSVD is not associated with functional connectivity alterations.^21,22^

The goal of the present study was twofold. Firstly, we aimed to investigate the effect of ageing on functional connectivity at older age, where we hypothesize that functional connectivity will be lower in older people. Secondly, we aimed to investigate the effects of CSVD manifestations on functional connectivity, including the presence of white matter (WM) lesions, microbleeds, lacunar infarcts and atrophy on MRI. In this respect, we investigated the hypotheses that CSVD lowers functional connectivity and that CSVD shows an interaction effect with age where CSVD increases age-related functional connectivity decline. To investigate these hypotheses, we studied functional connectivity of large-scale brain networks and whole brain graph theoretical network properties in a large sample of adults aged 75 years and older with varying degrees of CSVD burden.

## Materials and methods

### Participants and procedures

Participants were community-dwelling older adults that participated in an earlier study. We used the baseline data from the MRI substudy of the Discontinuation of Antihypertensive Treatment in the Elderly (DANTE) study Leiden, which has been described previously.^23^ This dataset contains data from more than two-hundred older adults at risk of developing CSVD due to their age and hypertensive status. Participants were included through general practices and eligible for participation in the DANTE study Leiden if they were 75 years or older, used antihypertensive medication for no other reason than hypertension, had a current systolic blood pressure ≤ 160 mm Hg, and had a mini mental state examination (MMSE) score between 21 and 27. Exclusion criteria included a history of stroke or transient ischemic attack, major cardiovascular disease including heart failure, or a clinical diagnosis of dementia. Participants were not allowed to participate in the MRI substudy if they had any MRI contraindications. The study was approved by the Medical Ethical Committee of the Leiden University Medical Center. Written informed consent was obtained from all participants.

At baseline, 236 participants underwent MR imaging for the DANTE MRI substudy. Afterwards, 16 participants were excluded due to incidental MRI findings. The present analyses required a Resting State functional MR image (RS-fMRI), which was present for 204 participants. Subsequently, 37 participants were excluded due to corrupted files (*n* = 4), lack of whole brain coverage (*n* = 11), excessive motion (i.e. ≥3 mm movement in any direction; *n* = 17), or missing data (on CSVD imaging features; *n* = 5), leaving 167 participants for the present analyses.

### Data acquisition and preprocessing

Participants were scanned at the Leiden University Medical Center on a 3-Tesla Philips Achieva MRI scanner (Philips Medical Systems, Best, the Netherlands) equipped with a standard 32-channel head coil. RS-fMRI was acquired with echo planar imaging with repetition time (TR) = 2200 ms, echo time (TE) = 30 ms, flip angle (FA) = 80°, field of view (FOV) = 220 × 220 × 113 mm, 38 slices with 10% interslice gap, resulting in a voxel size of 2.75 × 2.75 × 2.72 mm and total scan duration of 7 min 29 sec. Three-dimensional T1-weighted (3D-T1) structural images were acquired with TR = 9.7 ms, TE = 4.6 ms, FA = 8°, FOV = 224 × 177 × 168 mm, voxel size = 1.17 × 1.17 × 1.40 mm. Fluid-attenuated inversion recovery (FLAIR) images were acquired with TR = 11 000 ms, TE = 125 ms, FA = 90°, FOV = 220 × 176 × 137 mm, matrix size = 320 × 240, 25 transverse slices, 5 mm thick. T2*-weighted images were acquired with TR = 45 ms, TE = 31 ms, FA = 13°, FOV = 250 × 175 × 112 mm. T2-weighted images were acquired with TR = 4200 ms, TE = 80 ms, FA = 90°, FOV = 224 × 180 × 144 mm, matrix size = 448 × 320, 40 slices, 3.6 mm thick.

Preprocessing of RS-fMRI and 3D-T1 images was performed with FMRIB’s Software Library (FSL, version 6.0.4).^24^ Non-brain tissue was removed from structural images using the brain extraction tool of FSL.^25^ RS-fMRI images were preprocessed in multiple steps. Firstly, the fMRI Expert Analysis Tool (FEAT) was used to apply brain extraction,^25^ motion correction with MCFLIRT,^26^ and spatial smoothing with a Gaussian kernel of Full Width Half Maximum of 5 mm. Additionally, registration parameters were calculated by registering the RS-fMRI images to 3D-T1 images with Boundary-Based Registration using FLIRT,^26,27^ and registering 3D-T1 images to the 2 mm MNI152 standard space image (Montreal Neurological Institute, Montreal, QC, Canada) with non-linear registration using FNIRT^28^ with a warp resolution of 10 mm. Secondly, an independent component analysis (ICA)-based strategy for Automatic Removal of Motion Artifacts (ICA-AROMA)^29,30^ was used for further denoising. Thirdly, high-pass temporal filtering with a cut-of frequency of 0.01 Hz was applied using FEAT. Lastly, RS-fMRI images were warped into standard space using the registration parameters calculated before.

### Cerebral small vessel disease imaging features

Conventional imaging markers for CSVD include WMHs, lacunar infarcts, and CMBs.^16^ For each participant, these markers were scored according to methods described previously.^23^ In short, WMH volume was determined by extracting the WM from affine registered to MNI152 standard space FLAIR images using a standard WM template and customized threshold to identify hyperintense voxels. Resulting WMH masks were manually checked and edited for quality control. Additionally, regional distribution of WMHs was assessed by scoring the extent of deep WMHs, and anterior, posterior, and lateral periventricular WMHs using the Fazekas scale.^31^ Deep WMHs were scored 0 (absent), 1 (punctate foci), 2 (beginning confluence), or 3 (large confluent areas). Periventricular hyperintensities in each area were scored 0 (absent), 1 (caps or pencil-thin lining), 2 (smooth halo), or 3 (irregular periventricular signal extending into the deep WM). Lacunar infarcts were identified on 3D-T1, FLAIR, and T2-weighted images as parenchymal defects with similar intensities as CSF, diameter ≥3 mm and surrounded by increased signal intensity on T2-weighted and FLAIR images. CMBs were defined as focal areas of signal void on T2-weighted images that increased on T2*-weighted images. CMBs were scored separately for lobar and deep brain regions (e.g. basal ganglia).

Further, a composite score of CSVD burden on MRI was created as described previously.^32^ The total CSVD score ranged from 0 to 3, where points were awarded for each of the following features: high WMH load (WMHs volume was dichotomized on the median), presence of one or more lacunar infarcts, presence of two or more microbleeds (lobar or deep).

Considering CSVD is associated with brain atrophy,^16^ we additionally determined the gray matter (GM) volume. To calculate GM volume 3D-T1 images were segmented using FSL’s SIENAX.^33,34^ GM volume was determined from the resulting GM partial volume map and adjusted for head size by multiplying with a subject-specific scaling factor as calculated by SIENAX.

### Resting state functional MRI processing

To study functional connectivity, we applied two frequently used methods. First, we used standard RSN templates to study within-network functional connectivity in large-scale brain systems. Second, we used graph theoretical measures to quantify network properties of the whole brain network. This way, we gain a broad perspective on functional connectivity changes that may be associated with ageing and interference from CSVD-related pathology.

#### Resting state networks

Using ICA, the brain can be decomposed into a set of networks that are consistently and reliably identified across subjects, studies and study groups.^3,4,6^ Here, a network refers to a set of brain regions that are functionally connected, in other words: brain regions that show a high temporal correlation between the timeseries of all regions belonging to the network. Because of the nature of ICA, differences might occur in the decomposition of networks each time the algorithm is run resulting in slightly different components each time which can hamper the generalizability of the results and comparability to other studies. Therefore, ICA-derived standard network templates provided by Smith *et al*.^6^ were used to obtain consistent and reliable large-scale networks that can be compared across studies. These ten network templates represent the visual medial network, visual occipital network, visual lateral network, default mode network, cerebellar network, sensorimotor network, auditory network, executive control network, frontoparietal right network, and frontoparietal left network. Within-network functional connectivity was calculated using a dual regression approach as implemented in FSL. The ten RSN templates plus a CSF and WM template for nuisance regression were fed into the dual regression. Each subject’s preprocessed RS-fMRI dataset was first entered into a spatial regression to obtain subject-specific timeseries for each of the twelve templates (ten RSNs and CSF and WM templates). The subject-specific timeseries were then entered into a temporal regression to obtain subject-specific spatial maps of each RSN. These spatial maps contained a *z* score per voxel indicating how well the voxel fitted the specific RSN timeseries. For each RSN, mean *z* scores were calculated by averaging *z* scores of all voxels belonging to the RSN by overlaying a binary mask of the RSN template (thresholded at *z* = 3, similar to Smith *et al*.^6^) on the subject’s spatial maps. A low mean *z* score indicated that the within-network functional connectivity was low, whereas a high mean *z* score indicated that the within-network connectivity was high. The mean *z* score per RSN was used in subsequent statistical analyses.

#### Graph theoretical network measures

Graph theory is a mathematical approach that can be used to quantify aspects of the structure and dynamics of the brain as a complex network, modeling the elements of the network and the interactions between them.^35,36^ A network is defined as a graph consisting of nodes and edges, where nodes represent the elements of the network (small brain regions) and edges represent the interaction (in this case: the functional connectivity) between the nodes. The connectivity matrix is a different representation of the graph, where columns and rows represent the nodes and the matrix values represent the functional connectivity between nodes (edges), and can be used to calculate higher level summary measures that describe properties of the network such as integration and segregation.

GraphVar (version 2.03a)^37^ implemented in MATLAB (version 2020b) was used to construct connectivity matrices and calculate graph theoretical network measures. First, nodes were defined based on the functional parcellation of Seitzman *et al*.,^38^ an extension of the widely used Power *et al*.^39^ parcellation. The Seitzman parcellation has improved sampling of the subcortical regions and the cerebellum, which are often undersampled in other parcellation schemes, providing a more complete picture of the whole brain network. In total, 300 nodes (i.e. functional units) were defined by drawing spheres around MNI coordinates provided by Seitzman *et al*.^38^ As suggested by Seitzman *et al*.^38^ cortical spheres (*n* = 239) had a 5 mm radius, whereas subcortical (*n* = 34) and cerebellar (*n* = 27) spheres had a 4 mm radius. Second, mean BOLD timeseries were extracted for all 300 nodes for each subject from their preprocessed RS-fMRI scan. Prior to timeseries extraction from the nodes, nuisance regression was performed where mean timeseries of CSF and WM were regressed out of the data. Next, correlation coefficients (Pearson’s *r*) between mean timeseries of all nodes with all other nodes were calculated, resulting in a 300 × 300 connectivity matrix for each subject. The diagonal of the matrix, that is the correlation of the node with itself, was set to zero. Because the connectivity matrix is a fully connected matrix and this is unlikely (not all nodes will connect to all other nodes), a threshold needs to be applied to make the matrix sparser and remove spurious connections.^36^ Considering graph measures can be affected by the number of edges in a graph,^40^ a proportional threshold was applied. A proportional threshold controls the density of the matrix (e.g. maintain 5% of the strongest connections), ensuring the same number of edges are present in each subject. Since the choice of threshold value is arbitrary, it is advised to calculate graph measures across a range of thresholds.^36^ Thresholds ranging from 5% to 50% density with increments of 5% were investigated, resulting in ten connectivity matrices, one per threshold, for each subject. These matrices were used to calculate graph measures at each threshold for each subject. Statistical testing was performed at each threshold separately and the consistency of findings across thresholds was evaluated to ensure conclusions drawn from the results were not unduly influenced by an arbitrary threshold value.

Two frequently used graph measures were calculated that reflect properties of integration and segregation of the whole-brain network: weighted global efficiency (GE) and weighted clustering coefficient (CC). GE is a measure indicative of integration and provides information about how efficiently information can flow throughout the brain.^36,41,42^ CC is a measure indicative of segregation, the presence of specialized information processing clusters, and provides information about the cliquishness of the network.^36,42,43^ For each subject, both graph measures were calculated using the Brain Connectivity Toolbox^36^ implemented in GraphVar at all ten thresholds.

### Statistical analyses

SPSS (version 25.0) was used for all statistical analyses. Moderated hierarchical multiple linear regression models were constructed to assess the association between age and functional connectivity (both for RSNs connectivity and graph theoretical network measures) and the potential moderation of CSVD. Regression models were defined for each of the main CSVD features, that is WMH volume, lacunar infarcts, deep CMBs, lobar CMBs, and atrophy. In total, 150 regression models were calculated: for each RSN as the dependent variable (10 RSNs × 5 CSVD variables = 50 models) and for each of the graph theoretical network measures as the dependent variable (2 measures × 10 thresholds × 5 CSVD variables = 100 models). Each model consisted of three regression blocks. The first block contained age as the independent variable and sex and MMSE as covariates. In the second block, one of the CSVD features was added. In the third block, an interaction term of age with the CSVD feature was added. To prevent high collinearity in the models, continuous independent variables were mean centered (*x−x̄*) and centered variables were used to calculate the interaction terms. All variables were checked for normality beforehand and transformed using a natural log transformation if necessary. Tests of model assumptions were performed. Independence of observations was assessed using the Durbin-Watson statistic (≈2). Linearity and homoscedasticity were evaluated using plots of standardized residuals against standardized predicted values and partial plots (data points show a linear trend and are evenly distributed, no funnel or fan shape present). Absence of multicollinearity was assessed via the tolerance value (>0.1), variance inflation factor value (<10) and correlations among the variables (*r* < 0.7). Studentized deleted residuals (between -3 and 3), centered leverage values (<0.2), and Cook’s distance values (<1) were checked for unusual points. Normality of residuals was inspected via histograms (bell-shaped) and normal probability plots (data points center around the diagonal).

Besides these main analyses, subanalyses were performed as described in the Supplementary Materials. Associations between age and functional connectivity were tested in smaller age ranges to investigate potential different trajectories within late adulthood. Further, the four regional measures of WMH burden were used to examine regional associations of WMHs with functional connectivity. Next, analyses were performed using a composite CSVD score to assess the combined effect of CSVD features on the outcome. Lastly, voxelwise analyses were performed in the RSNs to explore whether regional associations were present within (sub)networks that may not be detectable on the whole network level.

For all analyses, a multiple comparison correction was applied for interpretation of the individual associations. For RSN analyses, a Bonferroni adjustment of 10 (number of RSNs) was applied, resulting in an adjusted significance threshold of 0.005. For the analyses of graph theoretical network measures a Bonferroni adjustment of 2 was applied, i.e. the number of graph measures calculated, resulting in an adjusted significance threshold of 0.025.

### Data availability

The data that support the findings of this study are available from the corresponding author, upon reasonable request.

## Results

### Demographic and clinical characteristics

Characteristics of the study sample are shown in Table 1. The mean age was 80.3 ± 3.8 years. All subjects presented with WMHs, with around one third showing severe WMHs (Fazekas score 3). Lacunar infarcts and CMBs were present in approximately a quarter of the sample. Approximately one fifth of the sample displayed medium to high CSVD burden.

**Table 1:** Characteristics of the study population (*n* = 167)

Characteristic		
**Demographic and clinical**		
Age, years	80.3	(3.8)
Sex, female	97	(58.1%)
>6 years of education ^a^	110	(65.9%)
Current smoking	13	(7.8%)
Alcohol (≥14 units per week)	16	(9.6%)
Diabetes mellitus	33	(19.8%)
Cardiovascular disease ^b^	21	(12.6%)
Hypertension	167	(100%)
Duration of antihypertensive treatment ^c^		
<1 year	4	(2.4%)
1–5 years	46	(27.5%)
>5 years	113	(67.7%)
Systolic blood pressure, mm Hg	145.4	(21.9)
Diastolic blood pressure, mm Hg	80.7	(11.2)
Pulse pressure, mm Hg	64.7	(15.8)
Mean arterial pressure, mm Hg	102.2	(13.7)
MMSE	26	(25–27)
**MRI features of CSVD**		
WMH volume, mL	20.1	(8.0–51.0)
WMH deep 0/1/2/3, %	-	50/28/17/5
WMH anterior periventricular 0/1/2/3, %	-	0/17/46/37
WMH posterior periventricular 0/1/2/3, %	-	0/26/38/36
WMH lateral periventricular 0/1/2/3, %	-	0/25/47/28
Lacunar infarct present	45	(26.9%)
Median number of lacunar infarct	1	(1–2)
Cerebral microbleeds present	41	(24.6%)
Deep microbleeds present	24	(14.4%)
Median number of deep microbleeds	1	(1–2.8)
Lobar microbleeds present	28	(16.8%)
Median number of lobar microbleeds	1	(1–2)
Gray matter volume, mL	632.4	(54.1)
Composite CSVD score		
0: no CSVD burden	64	(38.3%)
1: low CSVD burden	67	(40.1%)
2: medium CSVD burden	29	(17.4%)
3: high CSVD burden	7	(4.2%)

Data are presented as mean (*SD*), median (interquartile range), or number (percentage) where appropriate.

MMSE, Mini mental state examination, CSVD, Cerebral small vessel disease, WMH, White matter hyperintensities.

a . missing for *n* = 10 participants.

b . cardiovascular disease indicates myocardial infarction or a coronary intervention procedure >3 years ago or peripheral arterial disease.

c . missing for *n* = 4 participants.

### Functional connectivity within resting state networks

**Figure 1:**
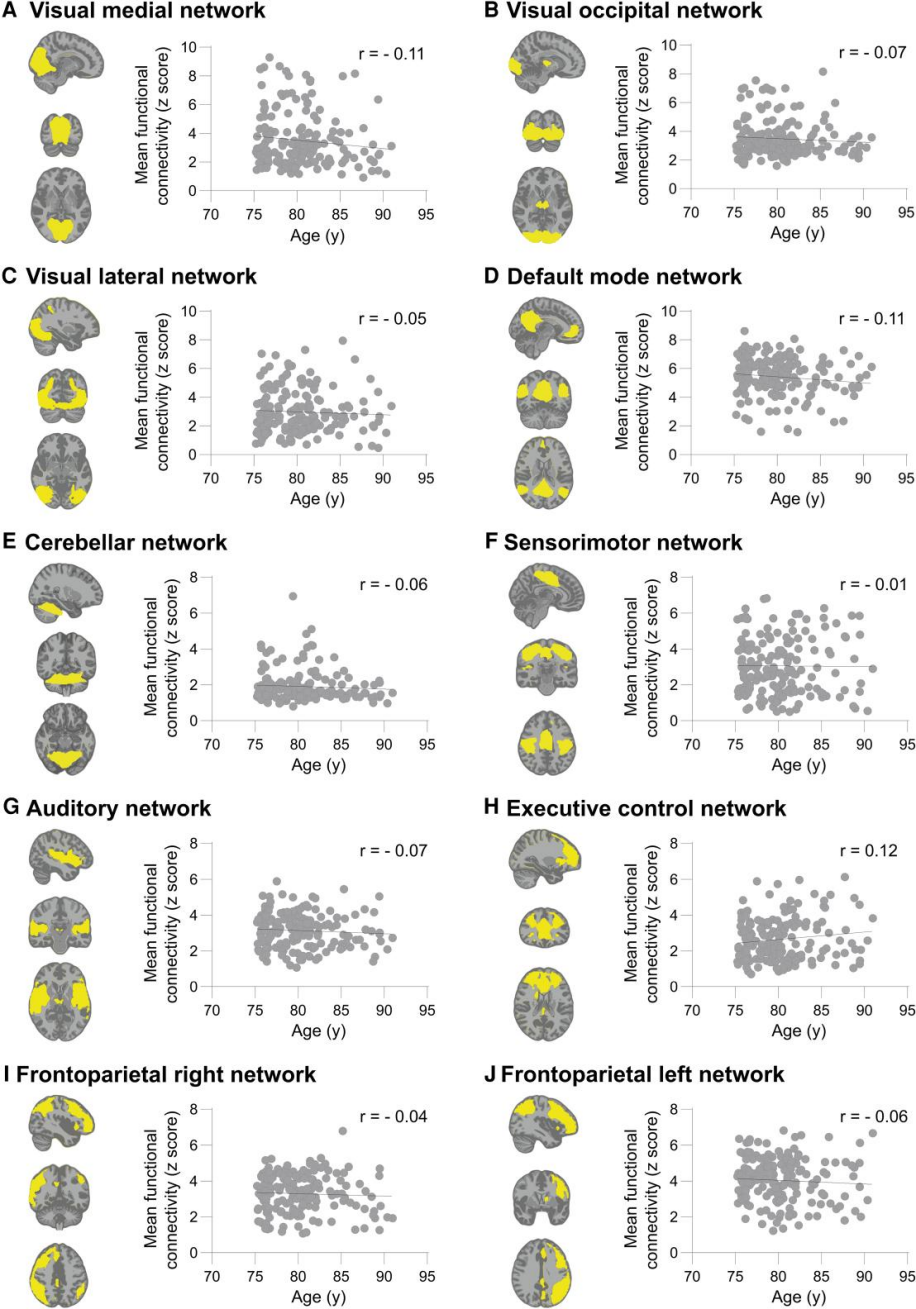
**Associations between age and functional connectivity within the resting state networks (RSNs).** The RSN is displayed in yellow, overlaid on the MNI standard anatomical brain image. Scatterplots show the association between age on the *x*-axis and mean functional connectivity within the RSN on the *y*-axis and include the least squares regression line. None of the associations were significant.

Associations between age and mean functional connectivity within each of the RSNs are shown in Table 2 and Fig. 1. The first column of Table 2 shows the standardized regression coefficients while Fig. 1 shows scatterplots of each RSN. All associations except for the executive control network demonstrated a downward slope, suggesting lower functional connectivity with increasing age. However, none of the associations were significant, indicating that within-network functional connectivity does not change with ageing in this sample of older adults (see Supplementary Table 1 for exact *P*-values). This finding is further supported by subanalyses that did not show any evidence of different trajectories across the age range. No significant linear associations within any of the smaller age ranges (Supplementary Table 4) and no quadratic relationships were found (Supplementary Table 6). Exploratory voxelwise analyses also showed no significant associations between age and regional connectivity within any of the networks (see Supplementary Materials).

**Figure 2:**
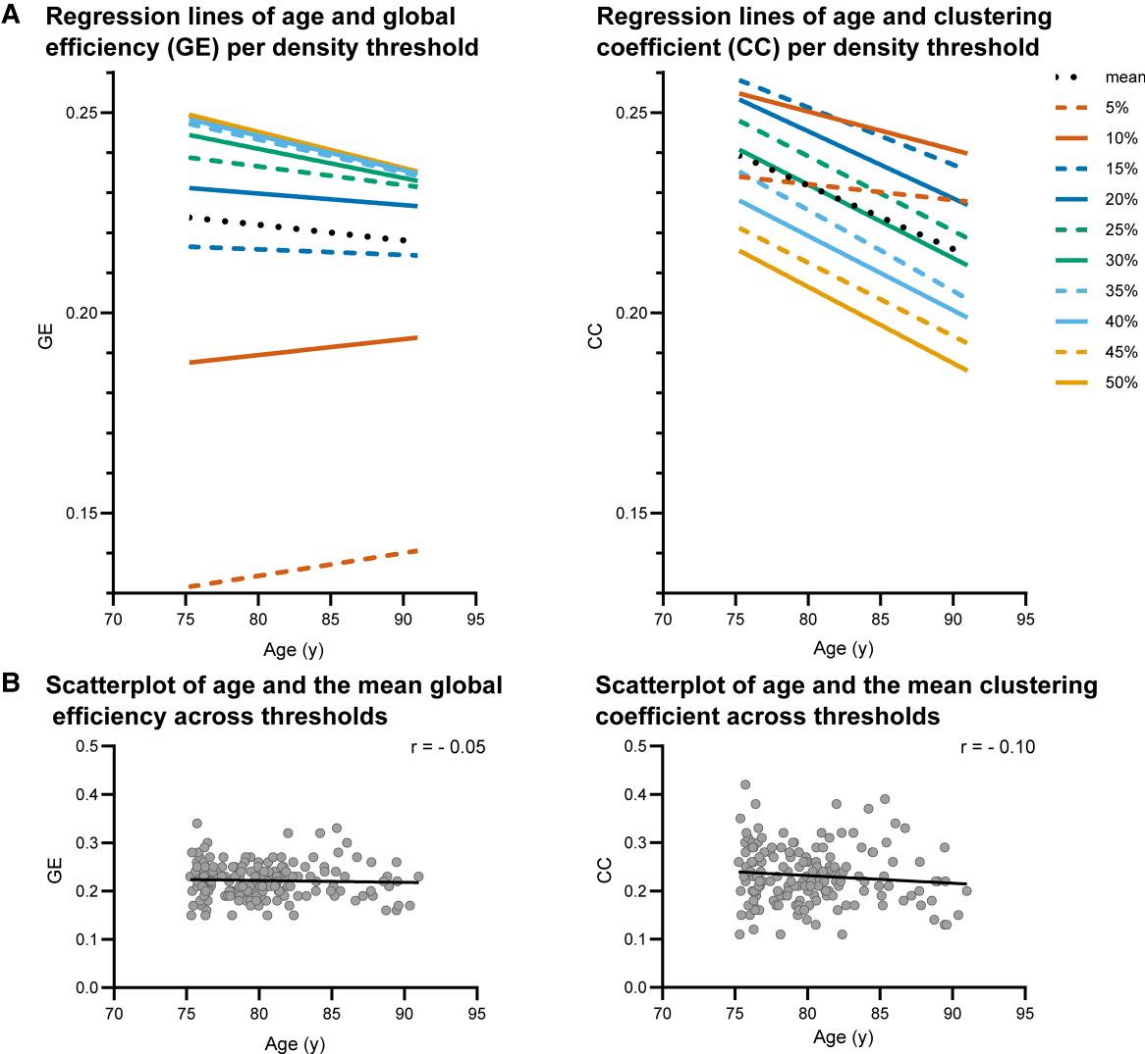
**Associations of age and graph theoretical network measures.** Panel A displays the least squares regression lines for the age and GE or age and CC relationships for each individual density threshold as well as the mean across all thresholds (for reference purposes to panel B) on an inflated *y*-axis. Each line represents 167 data points. Related standardized regression coefficients and *P*-values are reported in Table 3 and Supplementary Table 2, respectively. In the left graph, regression lines of 35–50% partially overlap. Panel B shows the scatterplot of age and the mean of the global efficiency across all thresholds (*r* = −0.05, *P* = 0.563) and the scatterplot of age and the mean of the clustering coefficient across all thresholds (*r* = −0.10, *P* = 0.179).

**Table 2:** Standardized regression coefficients (corrected for sex and MMSE) showing main and interaction effects of age and CSVD features on mean within-network functional connectivity for each resting state network

	Age	WMH volume		Lacunar infarcts		Deep CMBs		Lobar CMBs		GM volume	
RSN	ME	ME	IE	ME	IE	ME	IE	ME	IE	ME	IE
Visual medial	-0.143	0.079	-0.184	0.062	0.184	-0.103	0.058	-0.072	-0.041	-0.071	0.134
Visual occipital	-0.050	0.051	-0.122	0.163	0.061	-0.116	0.043	-0.074	-0.060	-0.095	0.179
Visual lateral	-0.058	0.083	-0.196	0.047	0.150	-0.052	-0.060	-0.118	-0.124	-0.034	0.131
Default mode	-0.108	-0.097	-0.062	0.130	0.018	-0.072	0.038	-0.056	0.062	0.084	-0.104
Cerebellar	-0.091	0.037	-0.023	0.084	0.206	-0.119	0.145	0.004	0.111	-0.112	0.022
Sensorimotor	-0.007	-0.003	-0.065	0.134	0.027	-0.185	0.022	-0.094	-0.043	-0.015	0.049
Auditory	-0.042	0.060	-0.010	0.096	0.017	-0.067	0.021	-0.015	-0.039	0.034	0.052
Executive control	0.096	-0.046	-0.036	0.016	0.050	-0.081	-0.059	-0.160	-0.036	-0.002	-0.031
Frontoparietal right	-0.018	0.071	0.063	0.002	0.196	0.068	-0.002	0.027	-0.023	0.037	0.032
Frontoparietal left	-0.038	0.053	-0.038	-0.021	0.071	0.014	0.064	-0.086	0.045	-0.036	-0.068

None of the associations were significant after multiple comparisons correction (Bonferroni corrected *P* < 0.005). Exact *P*-values are provided in Supplementary Table 1.

WMH, white matter hyperintensities; CMBs, cerebral microbleeds; GM, gray matter; CSVD, cerebral small vessel disease; RSN, resting state network; ME, main effect; IE, interaction effect.

Associations between CSVD features and mean within-network functional connectivity are also presented in Table 2. For each CSVD feature, the standardized regression coefficients of the main effect beyond age and interaction effect with age are presented. Exact *P*-values are provided in Supplementary Table 1. Nine out of 100 CSVD regression coefficients in Table 2 demonstrated an uncorrected *P* < 0.05 (*P*-values between 0.012 and 0.042), however none of the associations remained significant after correcting for multiple comparisons (*P* < 0.005). Additionally, no significant main or interaction effects were found for regional WMH measures (Supplementary Table 8) nor for the composite CSVD score (Supplementary Table 11). Exploratory voxelwise analyses also did not show any significant association between CSVD features and regional connectivity within any of the networks (see Supplementary materials), showing that CSVD does not affect within-network functional connectivity in our sample of older adults.

### Graph theoretical network measures

The results of the analyses between age and GE and between age and CC at all thresholds are shown by the standardized regression coefficients in the first columns of Table 3 and Table 4. Exact *P*-values are provided in Supplementary Tables 2 and 3. Figure 2A shows the regression lines of age and GE and CC across the threshold range—note that the *y*-axis is inflated. The regression lines at 5% and at 10% density thresholds deviate from the trend shown at other thresholds, indicating that these thresholds may be too stringent. For visualization purposes, the mean GE and mean CC across all thresholds were calculated and scatterplots with age are shown in Fig. 2B. Both GE and CC show a small, but non-significant decrease with advancing age. Subanalyses using age groups showed no significant linear association within smaller age ranges (Supplementary Table 5) and no quadratic relationships at any of the thresholds for both GE and CC (Supplementary Table 7). Consistency of findings across thresholds indicate findings were robust and not unduly influenced by the arbitrariness of threshold values.

**Table 3:** Standardized regression coefficients (corrected for sex and MMSE) showing main and interaction effects of age and CSVD features on global efficiency at each density threshold

	Age	WMH volume		Lacunar infarcts		Deep CMBs		Lobar CMBs		GM volume	
Density threshold	ME	ME	IE	ME	IE	ME	IE	ME	IE	ME	IE
5%	0.109	-0.102	-0.048	0.049	0.032	-0.087	-0.023	-0.084	-0.065	0.025	-0.109
10%	0.076	-0.087	-0.068	0.102	0.053	-0.100	0.038	-0.067	-0.027	0.058	-0.046
15%	0.022	-0.021	-0.034	0.130	0.063	-0.057	0.075	-0.032	-0.039	0.028	-0.021
20%	-0.007	0.004	-0.002	0.128	0.062	-0.034	0.081	-0.019	-0.051	0.021	-0.015
25%	-0.032	0.012	0.013	0.117	0.054	-0.037	0.091	-0.028	-0.045	0.015	-0.018
30%	-0.045	0.012	0.021	0.112	0.046	-0.039	0.096	-0.029	-0.039	0.018	-0.021
35%	-0.056	0.021	0.025	0.109	0.040	-0.044	0.103	-0.023	-0.054	0.012	-0.019
40%	-0.058	0.021	0.025	0.106	0.038	-0.044	0.103	-0.023	-0.052	0.013	-0.019
45%	-0.059	0.019	0.026	0.106	0.036	-0.045	0.102	-0.025	-0.050	0.015	-0.020
50%	-0.059	0.018	0.027	0.106	0.035	-0.045	0.102	-0.025	-0.049	0.016	-0.020

None of the associations were significant (Bonferroni corrected *P* < 0.025). Exact *P*-values are provided in Supplementary Table 2.

WMH, white matter hyperintensities; CMBs, cerebral microbleeds; GM, gray matter; CSVD, cerebral small vessel disease; ME, main effect; IE, interaction effect.

**Table 4:** Standardized regression coefficients (corrected for sex and MMSE) showing main and interaction effects of age and CSVD features on clustering coefficient at each density threshold

	Age	WMH volume		Lacunar infarcts		Deep CMBs		Lobar CMBs		GM volume	
Density threshold	ME	ME	IE	ME	IE	ME	IE	ME	IE	ME	IE
5%	-0.025	0.089	0.020	0.149	0.053	-0.025	0.088	0.030	-0.028	-0.061	-0.004
10%	-0.057	0.085	0.030	0.134	0.078	-0.014	0.103	0.026	-0.015	-0.023	0.023
15%	-0.077	0.092	0.021	0.132	0.054	-0.020	0.098	0.028	-0.020	-0.018	0.036
20%	-0.083	0.080	0.035	0.122	0.052	-0.023	0.096	0.013	-0.022	-0.008	0.026
25%	-0.092	0.069	0.038	0.107	0.045	-0.025	0.098	0.002	-0.019	-0.001	0.015
30%	-0.092	0.074	0.042	0.111	0.041	-0.030	0.108	0.003	-0.039	0.000	0.013
35%	-0.096	0.064	0.042	0.107	0.038	-0.033	0.104	-0.002	-0.037	0.009	0.006
40%	-0.096	0.055	0.042	0.105	0.032	-0.034	0.102	-0.007	-0.037	0.017	0.001
45%	-0.096	0.048	0.043	0.103	0.028	-0.036	0.099	-0.012	-0.037	0.024	-0.003
50%	-0.097	0.042	0.043	0.100	0.024	-0.037	0.096	-0.016	-0.037	0.032	-0.006

None of the associations were significant (Bonferroni corrected *P* < 0.025). Exact *P*-values are provided in Supplementary Table 3.

WMH, white matter hyperintensities; CMBs, cerebral microbleeds; GM, gray matter; CSVD, cerebral small vessel disease; ME, main effect; IE, interaction effect.

Furthermore, Tables 3 and 4 show the standardized regression coefficients of the main and interaction effects of CSVD on GE and CC, showing no significant associations at any threshold (see Supplementary Tables 2 and 3 for exact *P*-values). Also, no significant main or interaction effects were found at any threshold for the composite CSVD score (Supplementary Table 12). For regional WMH measures, deep WMHs showed an interaction with age that was significant for GE at the 10% threshold (*β* = -0.221, *P* = 0.004) and 15% threshold (*β* = -0.190, *P* = 0.014) (Supplementary Table 9), while no main or interaction effects were found for CC (Supplementary Table 10). Taken together, as most thresholds showed no significant effects, these findings indicate that CSVD does not affect GE or CC of the whole brain network.

## Discussion

In this study, we examined functional connectivity in adults aged 75 years and older and investigated whether CSVD as ageing morbidity influenced functional connectivity. In our sample of community-dwelling older adults, functional connectivity was stable across the age range and not affected by features of CSVD.

Although there is an extensive body of literature assessing ageing effects on functional connectivity,^10–15,44–49^ little is known about functional connectivity alterations in people beyond 75 years of age. Here, we investigated functional connectivity in 75 to 91 year olds and found no associations between age and functional connectivity within large-scale brain networks or graph theoretical measures of brain integration and segregation. Previous research indicated a general ageing trend of decreased within-network functional connectivity, increased integration and a loss of segregation of the brain (see Edde *et al*.^11^ and Jockwitz and Caspers^15^ for recent reviews). The present study extends the current literature by showing this trend is not present in the adults aged 75 years and older. Neither a linear nor a quadratic trend could be identified in this sample of older adults. Although it has been suggested that functional connectivity alterations might be strongest in people aged 65 years and above,^11,14^ our findings indicate a possibility of a plateauing effect of age on functional connectivity in the middle old and oldest old. It may be hypothesized that the largest age-related alterations in functional connectivity may occur in the early stage of late adulthood, whereas functional connectivity remains relatively unchanged in the latter stages of late adulthood. Still, brain connectivity in older persons could also be influenced by other factors such as different individual levels of vascular pathology.

In this study, we investigated whether CSVD—one of the most common age-related morbidities—is a factor in predicting functional connectivity in older persons. In our sample of 75 to 91 year olds, none of the features of CSVD, i.e. WMHs, lacunar infarcts, microbleeds, or atrophy, affected functional connectivity. Nevertheless, reduced functional connectivity was found in younger patients with CSVD (mean age of 65 years) in the default mode and frontoparietal networks.^20^ Liu *et al*.^20^ used a case-control setting whereas we investigated CSVD effects continuously within an older sample which might result in different findings. We found no associations between any of the CSVD features and functional connectivity in any of the ten networks.

In addition to our within-network findings, we did not find any significant associations between CSVD and graph theoretical network measures. Other studies that used comparable graph theoretical analyses^21,22^ also did not find an association between CSVD and functional connectivity in a younger population (mean age of 68 and 66 years). Neither differences in whole-brain properties of integration (GE) and segregation (CC) between CSVD patients and age-matched control subjects were found,^22^ nor was there an association between CSVD burden and both graph measures within a group of CSVD patients.^21^ Our data extend these findings by showing that also in older adults, no association between CSVD and brain network properties of integration and segregation was found.

This study has several strengths and limitations. The main strengths of our study are that we investigated brain connectivity within an age range that was previously not explored. Moreover, we investigated multiple aspects of functional connectivity, being functional connectivity within RSNs and graph theoretical network properties of the whole brain network. Inconsistencies in the literature can arise due to differences in methodology, including different decompositions of the brain into functional units and networks. The use of standardized templates ensures a consistent, replicable breakdown of the brain into smaller regions or networks that improves generalizability and comparability to other study samples, yet potentially result in more group-level noise in the analyses that might result in less reliable functional connectivity estimates in comparison with obtaining study-specific templates (e.g. via ICA). The generalizability of our findings is limited by the selection criteria of the DANTE Study Leiden. Although CSVD-related pathologies were present in our participants, excluding individuals with a history of serious cardiovascular events such as stroke or heart failure resulted in a relatively healthy sample of older persons. More than 60% of the study sample showed presence of CSVD-related pathology, where the CSVD burden was low for most participants. This is representative of the general population of community-dwelling older adults and in line with expectations as the DANTE Study Leiden did not target individuals with high CSVD burden specifically, but older adults at risk of developing CSVD due to their advanced age and hypertensive status. Implications include that there may have been a reduced possibility to detect significant associations between CSVD and functional connectivity. It could be that effects occurring in the earlier disease stages may have been too subtle to detect or effects occur at later disease stages that were obscured by the majority of participants having a low CSVD burden. An additional sample limitation is that all participants were hypertensive and experienced mild cognitive problems, limiting our ability to extrapolate our findings to all older persons in the general population. Since all participants were on hypertensive treatment, we could not examine potential hypertensive effects. Although our sample consisted of persons with mild cognitive deficits, the effect of cognitive bias in our sample was likely small since our results showed that there was no significant cognitive contribution in any of the associations studied. Lastly, the main radiological features of CSVD were included in this study, but also other vascular factors associated with CSVD such as enlarged perivascular spaces, cerebrovascular reactivity or blood-brain barrier dysfunction might be of interest to further explore CSVD and functional connectivity associations. Unfortunately, these data were not available for this cohort and remain an open area for future research.

## Supplementary Material

fcad126_Supplementary_Data

## Abbreviations

CC =: clustering coefficient
CMB =: cerebral microbleed
CSF =: cerebrospinal fluid
CSVD =: cerebral small vessel disease
fMRI =: functional magnetic resonance imaging
GE =: global efficiency
GM =: gray matter
ICA =: independent component analysis
IE =: interaction effect
ME =: main effect
MMSE =: mini mental state examination
RS-fMRI =: resting state functional MRI
RSN =: resting state network
WM =: white matter
WMH =: white matter hyperintensity
