# Supplementary material for: Functional connectivity in older adults—the effect of cerebral small vessel disease
Source: Brain Commun. 2023 Apr 19;5(3):fcad126. doi: 10.1093/braincomms/fcad126 (PMC10165246; doi:10.1093/braincomms/fcad126)
Supplement: fcad126_Supplementary_Data [file fcad126_supplementary_data.pdf]

SUPPLEMENTARY MATERIALS

Functional connectivity in older adults – the effect of cerebral small vessel disease

Nadieh Drenth[a], Jessica C. Foster-Dingley[a,b], Anne Suzanne Bertens[a,b], Nathaly Rius Ottenheim[b], Roos C. van der Mast[b,c], Serge A.R.B. Rombouts[a,d,e], Sanneke van Rooden[a], Jeroen van der Grond[a]

a. Department of Radiology, Leiden University Medical Center, Leiden, The Netherlands.

b. Department of Psychiatry, Leiden University Medical Center, Leiden, The Netherlands

c. Department of Psychiatry, Collaborative Antwerp Psychiatric Research Institute (CAPRI)–University of Antwerp, Antwerp, Belgium

d. Leiden University, Institute of Psychology, Leiden, The Netherlands.

e. Leiden Institute for Brain and Cognition, Leiden, The Netherlands.

1. Supplementary Methods

In addition to the main analyses described in the main text, subanalyses were performed to further explore associations between age, cerebral small vessel disease (CSVD) and functional connectivity.

1.1 Age groups

Rather than assessing the whole age-range continuously, subanalyses were performed using smaller age ranges to investigate potential different trajectories of the association between age and functional connectivity within late adulthood. The sample was split into three age groups: 75-79 years (n = 88), 80-84 years (n = 55), and 85 years and older (n = 24). Associations between age and mean functional connectivity within ten standard resting state networks (RSNs) and both graph theoretical measures were examined separately for each group using linear regression models. Further, quadratic relationships were tested using ANOVA models. For both models, the functional connectivity measure was entered as the dependent variable, age as the independent variable and sex and MMSE as covariates.

1.2 Regional distribution of WMHs

In addition to examining associations with whole brain white matter hyperintensity (WMH) burden (analyses with total WMH volume in the main text), distribution of WMHs across the brain were taken into account by separately assessing associations for deep WMHs, anterior-, lateral- and posterior periventricular WMHs. Moderated hierarchical multiple linear regression models were constructed for each of the regional WMH scores the same way as for

the other CSVD features described in the main text, paragraph 2.5. Both main effects and interaction effects with age were assessed.

1.3 CSVD composite score

A composite score of the main CSVD features (WMHs, lacunar infarcts, and cerebral microbleeds) might aid in the detection of the combined effect of CSVD on the outcome. Main and interaction effects of the composite CSVD score with age on functional connectivity were assessed using moderated hierarchical multiple linear regression models. The models were constructed in the same way as for the individual CSVD features as described in the main text, paragraph 2.5.

1.4 Voxelwise analyses in Resting State Networks

For the resting state networks, voxelwise analyses were performed to examine whether regional associations were present within (sub)networks that may not be detectable on the whole network level. General linear models were constructed and main effects of age and the CSVD features (WMHs volume, the 4 regional WMH measures, lacunar infarcts, deep cerebral microbleeds, lobar cerebral microbleeds, atrophy, and the composite CSVD score) as well as interaction effects were tested using FSL's randomise[1] with 5000 permutations per association. The resulting statistical maps were Family-Wise Error (FWE) corrected for multiple comparisons of all voxels by the Threshold-Free Cluster Enhancement technique (TFCE).[2]

2. Supplementary Results

2.1 Complementary *p*-values

Supplementary to the analyses results presented in Tables 2-4 in the main text, exact *p*-values of the analyses are provided in Supplementary Tables 1-3 for completeness.

2.2 Age groups

No significant linear associations were found within any of the smaller age ranges for the association of age with mean functional connectivity within ten standard RSNs (Supplementary Table 4) and with both graph theoretical measures (Supplementary Table 5). No quadratic relationships were found for RSNs (Supplementary Table 6), nor for graph theoretical measures (Supplementary Table 7).

2.3 Regional WMH associations

For regional WMH measures, no significant main or interaction effects were found for the RSNs (Supplementary Table 8). Deep WMHs showed an interaction with age that was significant for global efficiency at the 10% threshold (β = -0.221, p = 0.004) and 15% threshold (β = -0.190, p = 0.014), but not at the other thresholds (Supplementary Table 9). No significant main or interaction effects were found for clustering coefficient (Supplementary Table 10).

2.4 CSVD composite score

No significant main or interaction effects were found for the composite CSVD score on mean functional connectivity within the RSNs (Supplementary Table 11), nor on global efficiency or clustering coefficient (Supplementary Table 12).

2.5 Voxelwise analyses in Resting State Networks

No significant associations between age and regional connectivity within any of the networks were found.

Supplementary Table 1. *Exact p-values for main and interaction effects of age and CSVD features on mean within-network functional connectivity for each resting state network*

	Age	WMH volume		Lacunar infarcts		Deep CMBs		Lobar CMBs		GM volume	
RSN	ME	ME	IE	ME	IE	ME	IE	ME	IE	ME	IE
Visual medial	0.071	0.314	0.018	0.428	0.032	0.185	0.496	0.357	0.640	0.385	0.107
Visual occipital	0.529	0.518	0.122	0.039	0.480	0.141	0.612	0.348	0.490	0.248	0.031
Visual lateral	0.468	0.294	0.012	0.549	0.085	0.509	0.483	0.134	0.155	0.684	0.118
Default mode	0.173	0.219	0.432	0.097	0.836	0.359	0.656	0.477	0.479	0.307	0.210
Cerebellar	0.243	0.637	0.764	0.276	0.015	0.121	0.082	0.961	0.197	0.163	0.789
Sensorimotor	0.931	0.965	0.408	0.088	0.756	0.017	0.794	0.234	0.620	0.854	0.560
Auditory	0.596	0.441	0.894	0.218	0.844	0.387	0.807	0.845	0.656	0.676	0.528
Executive control	0.229	0.558	0.648	0.844	0.564	0.302	0.490	0.042	0.681	0.977	0.712
Frontoparietal right	0.823	0.359	0.416	0.976	0.022	0.385	0.985	0.725	0.788	0.652	0.697
Frontoparietal left	0.633	0.502	0.629	0.793	0.411	0.860	0.457	0.274	0.607	0.665	0.416

Note. None of *p*-values were below the significance threshold (Bonferroni corrected $p < 0.005$). Abbreviations: WMH = white matter hyperintensities, CMBs = cerebral microbleeds, GM = gray matter, CSVD = cerebral small vessel disease, RSN = resting state network, ME = main effect, IE = interaction effect.

Supplementary Table 2. *Exact p-values for main and interaction effects of age and CSVD features on global efficiency at each density threshold*

	Age	WMH volume		Lacunar infarcts		Deep CMBs		Lobar CMBs		GM volume	
Density threshold	ME	ME	IE	ME	IE	ME	IE	ME	IE	ME	IE
5%	0.158	0.182	0.532	0.525	0.707	0.254	0.784	0.277	0.445	0.751	0.181
10%	0.327	0.258	0.374	0.183	0.531	0.191	0.644	0.380	0.754	0.471	0.575
15%	0.778	0.789	0.660	0.090	0.452	0.462	0.374	0.676	0.652	0.725	0.802
20%	0.928	0.963	0.980	0.096	0.465	0.659	0.338	0.808	0.553	0.792	0.852
25%	0.679	0.878	0.868	0.127	0.526	0.632	0.279	0.713	0.603	0.849	0.829
30%	0.561	0.873	0.782	0.144	0.588	0.610	0.252	0.710	0.645	0.825	0.799
35%	0.471	0.781	0.744	0.156	0.640	0.563	0.217	0.764	0.531	0.883	0.817
40%	0.453	0.788	0.740	0.165	0.654	0.566	0.219	0.763	0.544	0.873	0.818
45%	0.447	0.801	0.730	0.165	0.670	0.558	0.221	0.748	0.558	0.855	0.810
50%	0.447	0.816	0.725	0.165	0.676	0.560	0.222	0.744	0.564	0.841	0.806

Note. None of *p*-values were below the significance threshold (Bonferroni corrected $p < 0.025$). Abbreviations: WMH = white matter hyperintensities, CMBs = cerebral microbleeds, GM = gray matter, CSVD = cerebral small vessel disease, ME = main effect, IE = interaction effect.

Supplementary Table 3. *Exact p-values for main and interaction effects of age and CSVD features on clustering coefficient at each density threshold*

	Age	WMH volume		Lacunar infarcts		Deep CMBs		Lobar CMBs		GM volume	
Density threshold	ME	ME	IE	ME	IE	ME	IE	ME	IE	ME	IE
5%	0.754	0.253	0.795	0.056	0.536	0.745	0.300	0.697	0.751	0.454	0.964
10%	0.465	0.274	0.697	0.082	0.359	0.853	0.223	0.739	0.863	0.780	0.779
15%	0.325	0.238	0.785	0.088	0.525	0.793	0.245	0.721	0.818	0.825	0.661
20%	0.285	0.300	0.648	0.113	0.543	0.765	0.254	0.863	0.799	0.918	0.751
25%	0.237	0.371	0.617	0.162	0.596	0.747	0.241	0.981	0.829	0.993	0.858
30%	0.235	0.339	0.589	0.148	0.625	0.700	0.197	0.970	0.649	0.996	0.877
35%	0.215	0.407	0.587	0.163	0.657	0.670	0.214	0.981	0.665	0.910	0.946
40%	0.216	0.472	0.582	0.170	0.701	0.657	0.224	0.929	0.666	0.828	0.990
45%	0.215	0.530	0.578	0.179	0.740	0.638	0.236	0.881	0.665	0.763	0.975
50%	0.207	0.587	0.571	0.190	0.775	0.626	0.248	0.833	0.661	0.688	0.940

Note. None of *p*-values were below the significance threshold (Bonferroni corrected $p < 0.025$). Abbreviations: WMH = white matter hyperintensities, CMBs = cerebral microbleeds, GM = gray matter, CSVD = cerebral small vessel disease, ME = main effect, IE = interaction effect.

Supplementary Table 4. *Standardized regression coefficients and p-values (corrected for sex and MMSE) of the age and mean within-network functional connectivity association in each age group for each resting state network*

	75 – 80 years		80 – 85 years		85+ years	
RSN	β	*p*	β	*p*	β	*p*
Visual medial	-0.116	0.306	-0.016	0.912	-0.231	0.308
Visual occipital	0.000	0.999	-0.091	0.526	-0.406	0.070
Visual lateral	0.017	0.882	-0.075	0.603	-0.235	0.276
Default mode	-0.095	0.399	-0.187	0.186	0.066	0.765
Cerebellar	-0.089	0.424	-0.032	0.818	-0.117	0.600
Sensorimotor	-0.130	0.248	0.082	0.566	-0.129	0.514
Auditory	0.057	0.613	-0.073	0.605	-0.078	0.718
Executive control	0.032	0.780	0.134	0.348	-0.256	0.261
Frontoparietal right	0.045	0.686	0.212	0.134	-0.324	0.098
Frontoparietal left	-0.137	0.217	-0.050	0.721	0.193	0.390

Note. None of the associations were significant (Bonferroni corrected $p < 0.005$). Abbreviation: RSN = resting state network.

Supplementary Table 5. *Standardized regression coefficients and p-values (corrected for sex and MMSE) of the association between age and the graph measures of global efficiency and clustering coefficient in each age group at each density threshold*

	Global efficiency						Clustering coefficient					
	75 – 80 years		80 – 85 years		85+ years		75 – 80 years		80 – 85 years		85+ years	
Density threshold	β	*p*	β	*p*	β	*p*	β	*p*	β	*p*	β	*p*
5%	0.104	0.345	0.073	0.608	0.007	0.973	-0.029	0.794	0.137	0.329	-0.319	0.158
10%	-0.019	0.861	0.123	0.371	-0.246	0.259	-0.131	0.239	0.174	0.212	-0.455	0.037
15%	-0.097	0.380	0.174	0.207	-0.336	0.126	-0.171	0.124	0.195	0.160	-0.460	0.036
20%	-0.135	0.219	0.179	0.194	-0.337	0.088	-0.187	0.090	0.192	0.165	-0.461	0.035
25%	-0.170	0.121	0.180	0.190	-0.390	0.078	-0.205	0.062	0.186	0.179	-0.452	0.040
30%	-0.191	0.081	0.182	0.185	-0.394	0.075	-0.176	0.110	0.163	0.240	-0.457	0.037
35%	-0.176	0.108	0.169	0.222	-0.398	0.071	-0.187	0.088	0.168	0.227	-0.456	0.037
40%	-0.181	0.099	0.171	0.215	-0.401	0.070	-0.194	0.076	0.166	0.230	-0.453	0.039
45%	-0.185	0.090	0.172	0.213	-0.401	0.069	-0.202	0.065	0.166	0.227	-0.450	0.041
50%	-0.187	0.086	0.171	0.214	-0.402	0.069	-0.209	0.056	0.165	0.230	-0.449	0.041

Note. None of the associations were significant (Bonferroni corrected $p < 0.025$).

Supplementary Table 6. *Results of ANOVAs testing quadratic associations between age and functional connectivity within each resting state network*

RSN	$F_{(1, 164)}$	p
Visual medial	3.418	0.066
Visual occipital	0.045	0.832
Visual lateral	0.273	0.602
Default mode	0.228	0.634
Cerebellar	7.122	0.008
Sensorimotor	0.569	0.452
Auditory	0.131	0.717
Executive control	0.987	0.322
Frontoparietal right	2.682	0.103
Frontoparietal left	0.001	0.970

Note. None of the associations were significant (Bonferroni corrected $p < 0.005$). Abbreviation: RSN = resting state network.

Supplementary Table 7. *Results of ANOVAs testing quadratic associations between age and functional connectivity for the graph measures of global efficiency and clustering coefficient at each density threshold*

	Global efficiency		Clustering coefficient	
Density threshold	$F_{(1, 164)}$	p	$F_{(1, 164)}$	p
5%	0.570	0.451	0.870	0.352
10%	0.021	0.886	0.925	0.338
15%	0.243	0.622	1.133	0.289
20%	0.460	0.498	1.015	0.315
25%	0.369	0.544	0.771	0.381
30%	0.265	0.608	1.189	0.277
35%	0.457	0.500	0.946	0.332
40%	0.412	0.522	0.805	0.371
45%	0.357	0.551	0.656	0.419
50%	0.339	0.561	0.560	0.455

Note. None of the associations were significant (Bonferroni corrected $p < 0.025$).

Supplementary Table 8. *Standardized regression coefficients and p-values (corrected for sex and MMSE) showing main and interaction effects of age and regional WMH measures on mean within-network functional connectivity for each resting state network*

	Deep WMHs				AP WMHs				LP WMHs				PP WMHs			
	ME		IE		ME		IE		ME		IE		ME		IE	
RSN	β	*p*	β	*p*	β	*p*	β	*p*	β	*p*	β	*p*	β	*p*	β	*p*
Visual medial	-0.074	0.355	-0.178	0.024	-0.009	0.905	-0.059	0.454	-0.099	0.202	-0.127	0.104	0.003	0.965	0.029	0.716
Visual occipital	0.026	0.745	-0.170	0.034	0.064	0.425	-0.073	0.357	0.054	0.492	-0.064	0.422	0.026	0.742	-0.001	0.993
Visual lateral	-0.028	0.729	-0.198	0.013	0.024	0.768	-0.117	0.138	-0.050	0.525	-0.172	0.029	-0.072	0.365	-0.080	0.311
Default mode	-0.112	0.160	-0.126	0.112	-0.068	0.391	-0.087	0.267	-0.113	0.147	-0.048	0.543	-0.139	0.076	-0.021	0.791
Cerebellar	-0.012	0.875	-0.063	0.426	0.033	0.669	-0.046	0.554	-0.004	0.958	-0.061	0.432	-0.002	0.982	-0.014	0.861
Sensorimotor	-0.095	0.233	-0.149	0.060	-0.018	0.822	-0.004	0.960	-0.062	0.431	-0.049	0.535	-0.048	0.545	0.036	0.651
Auditory	-0.010	0.901	-0.090	0.257	0.039	0.622	-0.039	0.622	-0.017	0.822	-0.069	0.379	0.013	0.863	0.065	0.405
Executive control	-0.010	0.903	-0.108	0.176	0.045	0.572	-0.067	0.396	-0.036	0.643	-0.026	0.739	0.017	0.828	0.031	0.698
Frontoparietal right	-0.213	0.007	-0.048	0.538	-0.059	0.455	0.031	0.690	-0.021	0.792	0.054	0.493	-0.076	0.331	0.022	0.776
Frontoparietal left	-0.173	0.029	-0.075	0.342	-0.024	0.765	-0.158	0.045	-0.055	0.486	-0.078	0.325	-0.124	0.114	0.026	0.736

Note. None of the associations were significant after multiple comparisons correction (Bonferroni corrected $p < 0.005$). Abbreviations: WMH = white matter hyperintensities, AP = anterior periventricular, LP = lateral periventricular, PP = posterior periventricular, RSN = resting state network, ME = main effect, IE = interaction effect.

Supplementary Table 9. *Standardized regression coefficients and p-values (corrected for sex and MMSE) showing main and interaction effects of age and regional WMH measures on global efficiency at each density threshold*

	Deep WMHs				AP WMHs				LP WMHs				PP WMHs			
	ME		IE		ME		IE		ME		IE		ME		IE	
Density threshold	β	*p*	β	*p*	β	*p*	β	*p*	B	*p*	β	*p*	β	*p*	β	*p*
5%	-0.104	0.182	-0.133	0.086	-0.059	0.445	-0.071	0.354	-0.134	0.080	-0.080	0.298	-0.051	0.506	0.034	0.656
10%	-0.107	0.169	**-0.221**	**0.004**	-0.082	0.291	-0.096	0.211	-0.144	0.058	-0.071	0.350	-0.077	0.315	0.045	0.554
15%	-0.092	0.242	**-0.190**	**0.014**	-0.042	0.589	-0.049	0.530	-0.088	0.252	-0.032	0.678	-0.056	0.465	0.085	0.267
20%	-0.085	0.276	-0.158	0.042	-0.022	0.783	-0.011	0.886	-0.064	0.407	-0.011	0.889	-0.054	0.485	0.111	0.148
25%	-0.075	0.339	-0.134	0.084	-0.010	0.896	0.003	0.967	-0.049	0.520	-0.001	0.994	-0.057	0.460	0.128	0.093
30%	-0.069	0.377	-0.124	0.109	-0.004	0.955	0.016	0.834	-0.041	0.590	0.005	0.949	-0.055	0.471	0.133	0.080
35%	-0.070	0.370	-0.123	0.115	-0.001	0.986	0.010	0.901	-0.044	0.564	0.005	0.949	-0.060	0.435	0.138	0.070
40%	-0.069	0.376	-0.121	0.119	0.000	0.996	0.012	0.880	-0.042	0.581	0.006	0.941	-0.060	0.433	0.139	0.068
45%	-0.068	0.384	-0.121	0.120	0.000	0.996	0.014	0.853	-0.040	0.597	0.006	0.938	-0.059	0.438	0.140	0.067
50%	-0.068	0.387	-0.121	0.120	0.000	1.000	0.015	0.841	-0.040	0.601	0.006	0.939	-0.060	0.433	0.140	0.066

Note. Boldface indicates significance after multiple comparisons correction (Bonferroni corrected $p < 0.025$). Abbreviations: WMH = white matter hyperintensities, AP = anterior periventricular, LP = lateral periventricular, PP = posterior periventricular, ME = main effect, IE = interaction effect.

Supplementary Table 10. *Standardized regression coefficients and p-values (corrected for sex and MMSE) showing main and interaction effects of age and regional WMH measures on clustering coefficient at each density threshold*

	Deep WMHs				AP WMHs				LP WMHs				PP WMHs			
	ME		IE		ME		IE		ME		IE		ME		IE	
Density threshold	β	*p*	β	*p*	β	*p*	β	*p*	β	*p*	β	*p*	β	*p*	β	*p*
5%	-0.083	0.296	-0.101	0.201	-0.010	0.899	0.022	0.781	0.001	0.990	0.003	0.968	-0.022	0.777	0.118	0.130
10%	-0.054	0.495	-0.110	0.164	0.007	0.931	0.032	0.686	0.005	0.950	0.023	0.772	-0.022	0.778	0.125	0.105
15%	-0.046	0.564	-0.118	0.133	0.025	0.752	0.027	0.729	0.016	0.834	0.005	0.946	-0.022	0.780	0.116	0.135
20%	-0.046	0.558	-0.102	0.193	0.026	0.743	0.037	0.635	0.011	0.889	0.016	0.837	-0.028	0.713	0.127	0.099
25%	-0.047	0.545	-0.093	0.232	0.020	0.800	0.040	0.604	0.005	0.947	0.015	0.848	-0.038	0.625	0.132	0.086
30%	-0.052	0.508	-0.095	0.226	0.015	0.843	0.031	0.691	-0.010	0.897	0.024	0.758	-0.044	0.572	0.144	0.060
35%	-0.056	0.478	-0.095	0.225	0.012	0.882	0.031	0.687	-0.016	0.840	0.024	0.756	-0.050	0.515	0.146	0.056
40%	-0.057	0.463	-0.093	0.232	0.007	0.931	0.031	0.687	-0.023	0.761	0.025	0.744	-0.056	0.466	0.147	0.054
45%	-0.058	0.455	-0.093	0.232	0.004	0.958	0.032	0.674	-0.028	0.710	0.027	0.727	-0.060	0.437	0.148	0.052
50%	-0.059	0.450	-0.092	0.235	0.003	0.973	0.033	0.672	-0.032	0.679	0.028	0.712	-0.063	0.407	0.150	0.049

Note. None of the associations were significant after multiple comparisons correction (Bonferroni corrected $p < 0.025$). Abbreviations: WMH = white matter hyperintensities, AP = anterior periventricular, LP = lateral periventricular, PP = posterior periventricular, ME = main effect, IE = interaction effect.

Supplementary Table 11. *Standardized regression coefficients and p-values (corrected for sex and MMSE) showing main and interaction effects of age and the composite CSVD score on mean within-network functional connectivity for each resting state network*

	Composite CSVD score			
	ME		IE	
RSN	β	*p*	β	*p*
Visual medial	0.015	0.851	-0.018	0.815
Visual occipital	0.037	0.637	-0.039	0.623
Visual lateral	-0.013	0.870	-0.066	0.402
Default mode	0.003	0.970	-0.039	0.623
Cerebellar	0.010	0.900	0.087	0.262
Sensorimotor	0.004	0.958	-0.064	0.416
Auditory	0.051	0.509	-0.011	0.889
Executive control	-0.097	0.215	-0.005	0.947
Frontoparietal right	0.096	0.216	0.105	0.175
Frontoparietal left	0.011	0.893	0.029	0.711

Note. None of the associations were significant after multiple comparisons correction (Bonferroni corrected $p < 0.005$). Abbreviations: RSN = resting state network, ME = main effect, IE = interaction effect.

Supplementary Table 12. *Standardized regression coefficients and p-values (corrected for sex and MMSE) showing main and interaction effects of age and the composite CSVD score on the graph measures of global efficiency and clustering coefficient at each density threshold*

	Global efficiency				Clustering coefficient			
	ME		IE		ME		IE	
Density threshold	β	*p*	β	*p*	β	*p*	β	*p*
5%	-0.098	0.200	-0.072	0.346	0.097	0.210	0.025	0.749
10%	-0.049	0.522	-0.048	0.529	0.100	0.193	0.056	0.470
15%	0.020	0.793	-0.008	0.915	0.107	0.165	0.038	0.626
20%	0.041	0.597	0.009	0.908	0.092	0.231	0.047	0.544
25%	0.039	0.613	0.021	0.785	0.079	0.302	0.048	0.535
30%	0.038	0.620	0.024	0.750	0.083	0.282	0.048	0.530
35%	0.040	0.599	0.027	0.725	0.075	0.329	0.046	0.552
40%	0.039	0.609	0.027	0.727	0.069	0.370	0.043	0.576
45%	0.039	0.613	0.026	0.732	0.063	0.407	0.040	0.598
50%	0.038	0.618	0.026	0.733	0.058	0.447	0.038	0.620

Note. None of the associations were significant (Bonferroni corrected $p < 0.025$). Abbreviations: ME = main effect, IE = interaction effect.

References

1. Winkler AM, Ridgway GR, Webster MA, Smith SM, Nichols TE. Permutation inference for the general linear model. *NeuroImage*. 2014;92:381-397. doi:10.1016/j.neuroimage.2014.01.060

2. Smith SM, Nichols TE. Threshold-free cluster enhancement: addressing problems of smoothing, threshold dependence and localisation in cluster inference. *NeuroImage*. 2009;44(1):83-98. doi:10.1016/j.neuroimage.2008.03.061
